# Specifications of the ACMG/AMP Variant Classification Guidelines for Germline *DICER1* Variant Curation

**DOI:** 10.1155/2023/9537832

**Published:** 2023-03-29

**Authors:** Jessica N. Hatton, Megan N. Frone, Hannah C. Cox, Stephanie B. Crowley, Susan Hiraki, Noriko N. Yokoyama, Noura S. Abul-Husn, James F. Amatruda, Michael J. Anderson, Xavier Bofill-De Ros, Ann G. Carr, Elizabeth C. Chao, Kenneth S. Chen, Shuo Gu, Cecilia Higgs, Jerry Machado, Deborah Ritter, Kris Ann P. Schultz, Emily R. Soper, Mona K. Wu, Jessica L. Mester, Jung Kim, William D. Foulkes, Leora Witkowski, Douglas R. Stewart

**Affiliations:** ^1^Clinical Genetics Branch, Division of Cancer Epidemiology and Genetics, National Cancer Institute, Rockville, Maryland, USA; ^2^PreventionGenetics LLC, Marshfield, Wisconsin, USA; ^3^Invitae Corporation, San Francisco, California, USA; ^4^GeneDx, Gaithersburg, Maryland, USA; ^5^Ambry Genetics, Aliso Viejo, California, USA; ^6^Institute for Genomic Health, Icahn School of Medicine at Mount Sinai, New York, New York, USA; ^7^Cancer and Blood Disease Institute, Children's Hospital Los Angeles, Keck School of Medicine, University of Southern California, Los Angeles, California, USA; ^8^RNA Biology Laboratory, Center for Cancer Research, National Cancer Institute, Frederick, Maryland, USA; ^9^Weststat, Inc., Rockville, Maryland, USA; ^10^Division of Genetics and Genomics, Department of Pediatrics, University of California, Irvine, California, USA; ^11^Department of Pediatrics, University of Texas Southwestern Medical Center, Dallas, Texas, USA; ^12^Exact Sciences Laboratories, Madison, Wisconsin, USA; ^13^Baylor College of Medicine, Houston, Texas, USA; ^14^Cancer and Blood Disorders, Children's Minnesota, International Pleuropulmonary Blastoma/DICER1 Registry, Minneapolis, Minnesota, USA; ^15^Department of Human Genetics, McGill University, Montreal, Quebec, Canada

## Abstract

Germline pathogenic variants in *DICER1* predispose individuals to develop a variety of benign and malignant tumors. Accurate variant curation and classification are essential for reliable diagnosis of *DICER1-*related tumor predisposition and the identification of individuals who may benefit from surveillance. Since 2015, most labs have followed the American College of Medical Genetics and Genomics and the Association for Molecular Pathology (ACMG/AMP) sequence variant classification guidelines for *DICER1* germline variant curation. However, these general guidelines lack gene-specific nuances and leave room for subjectivity. Consequently, a group of *DICER1* experts joined ClinGen to form the *DICER1* and miRNA-Processing Genes Variant Curation Expert Panel (VCEP) to create *DICER1-*specific ACMG/AMP guidelines for germline variant curation. The VCEP followed the FDA-approved ClinGen protocol for adapting and piloting these guidelines. A diverse set of 40 *DICER1* variants were selected for piloting, including 14 known pathogenic/likely pathogenic (P/LP) variants, 12 known benign/likely benign (B/LB) variants, and 14 variants classified as variants of uncertain significance (VUS) or with conflicting interpretations in ClinVar. Clinically meaningful classifications (i.e., P, LP, LB, or B) were achieved for 82.5% (33/40) of the pilot variants, with 100% concordance among the known P/LP and known B/LB variants. Half of the VUS or conflicting variants were resolved with four variants classified as LB and three as LP. These results demonstrate that the *DICER1-*specific guidelines for germline variant curation effectively classify known pathogenic and benign variants while reducing the frequency of uncertain classifications. Individuals and labs curating *DICER1* variants should consider adopting this classification framework to encourage consistency and improve objectivity.

## 1. Introduction

The *DICER1* gene (NM_177438.3) is located on chromosome 14q32.13 and contains 27 exons encoding 1,922 amino acids. Germline pathogenic variation in *DICER1* is associated with an increased risk for the development of tumors in childhood and adulthood (OMIM # 601200) [1–3]. The DICER1 protein is an endoribonuclease that converts a hairpin-shaped miRNA precursor (pre-miRNA) to a mature miRNA duplex by removing the terminal loop. The RNase IIIa and RNase IIIb domains of DICER1 form two catalytic cores [4], cleaving at the 3′ and 5′ sides of the terminal loop, respectively, which are required to generate miRNAs derived from the 3p-arm (3p miRNAs) and 5p-arm (5p miRNAs) of the pre-miRNA accordingly.

*DICER1-*related tumor predisposition was first described in families with pleuropulmonary blastoma, a rare pediatric lung tumor [3]. The phenotypic spectrum has since expanded to include a wide range of benign and malignant neoplasms in both children and adults such as Sertoli-Leydig cell tumors, cervical and ovarian embryonal rhabdomyosarcoma, Wilms tumor, nasal chondromesenchymal hamartoma, pituitary blastoma, pineoblastoma, thyroid lesions, and other rare sarcomas [1, 5]. Surveillance recommendations aimed at early tumor detection exist for those with *DICER1-*related tumor predisposition due to germline variants in *DICER1* [6–8].

Germline variant classification relies on the weighing of many pieces of evidence, such as functional data, population frequency, clinical phenotype, and family segregation data. In 2015, the American College of Medical Genetics and Genomics (ACMG) and the Association for Molecular Pathology (AMP) issued a joint publication of standards and guidelines for the classification of germline sequence variants [9] as a starting point to standardize variant classification procedures. The Clinical Genome Resource (ClinGen) [10], a National Institutes of Health- (NIH-) funded resource aimed at further refining and centralizing gene and variant curation processes, has since created a number of variant curation expert panels (VCEPs) [11] that follow the Food and Drug Administration- (FDA-) recognized guidance for Public Human Genetic Variant Databases and the ClinGen Expert Panel process to tailor and pilot gene-specific modifications of the ACMG/AMP variant curation guidelines [12–15].

The ClinGen *DICER1* and miRNA-Processing Gene VCEP, hereafter referred to as the *DICER1* VCEP, were formed with the goal of developing such tailored germline sequence variant curation guidelines for *DICER1* and eventually other miRNA-processing genes associated with inherited syndromes (https://clinicalgenome.org/affiliation/50050/). Here, we describe the process of our VCEP formation, the evidence code specification for *DICER1*, and pilot curation.

## 2. Methods

In 2019, a variety of *DICER1* experts from across North America convened virtually to form the *DICER1* VCEP (https://clinicalgenome.org/affiliation/50050/), following the ClinGen VCEP protocol (https://clinicalgenome.org/site/assets/files/3635/variant_curation_expert_panel_vcep_protocol_version_9-2_3.pdf). Membership included clinicians, basic scientists, laboratory geneticists, and variant scientists. Initially, 22 group members were divided into four subgroups (phenotype, penetrance, computational, and functional) to critically assess and modify a subset of the ACMG/AMP variant curation evidence codes for *DICER1*-specific germline variant curation. A preliminary set of specifications was defined in November 2020 using MANE transcripts NM_177438.2 and MONDO:0017288.

The specifications were piloted on 40 *DICER1* variants with submissions in ClinVar. These included 14 known pathogenic or likely pathogenic (P/LP) variants, 12 known benign or likely benign (B/LB) variants, and 14 variants with conflicting interpretations or classified as variants of uncertain significance (VUS). Classifications reflect ClinVar submissions as of November 2020 except for two of the P/LP variants which were updated more recently due to a known incongruence between one laboratory's ClinVar submissions (VUS) and internal classifications (LP) at the time of the data pull. Pilot variants were intentionally selected such that missense, nonsense, frameshift, synonymous, and intronic variants were represented, giving the opportunity to test the performance of as many evidence codes as possible. Each variant was double-curated by two of the six biocurators to ensure evidence codes were being interpreted and applied uniformly. All biocurators had prior variant curation experience through other ClinGen VCEPs and/or employment at a commercial genetic testing laboratory offering clinical genetic testing for the *DICER1* gene. In addition to published cases, relevant internal case-level data stripped of personally identifiable information was obtained by VCEP members working at testing laboratories, clinics, and the pleuropulmonary blastoma/*DICER1* Registry (http://www.ppbregistry.org, NCT03382158) using an organized spreadsheet guide. Variants were curated within the ClinGen Variant Curation Interface [16]. Final classifications were determined according to the original evidence code combinations [9] plus a handful of predetermined combinations supported by a Bayesian framework [17]. In cases of conflicting benign and pathogenic evidence codes, a Bayesian point system was employed to reach a final classification [18]. Evidence codes were further adapted as appropriate during the pilot, and the final specifications were approved by the ClinGen Sequence Variant Interpretation (SVI) Committee in May 2022.

Our ACMG/AMP specifications will be updated periodically; to find the most current information, please visit https://clinicalgenome.org/affiliation/50050/ or https://cspec.genome.network/cspec/ui/svi/doc/GN024.

## 3. Results

### 3.1. *DICER1*-Specific Variant Curation Criteria

The *DICER1* VCEP specifications to the ACMG/AMP variant curation criteria are summarized in Table 1. Eight evidence codes (PM3, PM6, PP2, PP5, BP1, BP3, BP5, and BP6) were excluded due to redundancy, irrelevance with respect to *DICER1*, or published ClinGen guidance [19]. The remaining 20 criteria were kept with clarifications and/or gene-specific modifications to strength or scope.

### 3.2. Population Data (BA1, BS1, and PM2)

#### 3.2.1. *BA1* and *BS1*

BA1 is standalone, and BS1 is strong evidence for benign variation based on the frequency of a variant in the general population. To determine frequency cutoffs, the VCEP first calculated a realistic maximum allele frequency for a pathogenic *DICER1* variant using the Whiffin-Ware equation: maximum credible population allele frequency = disease prevalence × maximum allelic contribution/disease penetrance [20]. Disease prevalence was set to 1 in 10,600 people (1 in 21,200 alleles) based on estimates from population databases [21]. The maximum allelic contribution was set to 0.07 based on the proportion of the most common P/LP *DICER1* variant from Invitae internal data. Disease penetrance was set to 0.1 (i.e., 10%) based on the lower end of published penetrance estimates for individuals aged 50-60 years [22]. The resulting frequency, 0.00003, was conservatively increased by one order of magnitude for a BS1 cutoff of 0.0003 and another order of magnitude for a BA1 cutoff of 0.003. The VCEP chose to use noncancer gnomAD subpopulations to minimize the inclusion of cases. Generally, the most recent version of gnomAD with a noncancer subpopulation should be used. However, earlier versions should be considered relevant (e.g., superior sample size). Per published guidance, continental subpopulations must have greater than 2,000 alleles tested and a minimum of five alleles present [23].

#### 3.2.2. *PM2*

The PM2 criterion is intended to provide evidence of pathogenicity for variants that are absent from population databases or present only at low levels. The VCEP identified 19 P/LP or putative loss of function *DICER1* variants in noncancer gnomAD at low frequencies and expects that more will inevitably be present as databases grow. For this reason, the VCEP chose to establish a PM2 cutoff rather than to require absence. Based on the data from those 19 variants, the VCEP elected to apply PM2 for variants with a frequency less than 0.000005 across noncancer gnomAD with no more than one allele in any subpopulation and at least 20x coverage for that region of the gene in gnomAD. Such conditions would allow PM2 application for 15 of the 19 variants described previously. Per ClinGen SVI recommendations, PM2 should only be applied at a supporting level (https://www.clinicalgenome.org/site/assets/files/5182/pm2_-_svi_recommendation_-_approved_sept2020.pdf).

### 3.3. Computational and Predictive Data (PVS1, PS1, PM1, PM4, PM5, PP3, BP4, and BP7)

#### 3.3.1. *PVS1*

PVS1 provides very strong evidence of pathogenicity for null variants in a gene where the loss of function is a known mechanism of disease. This code is particularly relevant to *DICER1*, as most germline causative alleles are loss of function [1, 24]. The VCEP adopted previously published recommendations for the PVS1 application [25] but provided *DICER1-*specific details to simplify the application such as the nonsense-mediated decay cutoff and which exons, if skipped, would result in in-frame deletions. Notably, the VCEP deviated from the typical recommendation by precluding PVS1 application for start codon variants, as the p.Met1 site is not highly conserved in *DICER1*, and there are three possible in-frame alternate methionine residues at p.Met11, p.Met17, and p.Met24. Furthermore, internal lab data showed that, in multiple individuals, p.Met1 variants are not associated with any *DICER1* phenotype. A *DICER1-*specific PVS1 flowchart is provided in Supplementary Figure 1.

#### 3.3.2. *PP3* and *BP4*

PP3 and BP4 support level evidence codes based on computational predictors. The VCEP assessed the performance of several computational predictors, including metaSVM, CADD, BayesDel, and REVEL, on 15 known P/LP and 27 known B/LB *DICER1* missense variants. The best separation was attained using REVEL, a computational meta-predictor whose score reflects 13 individual computational tools [26]. Attempts were made to trichotomize REVEL score cutoffs for PP3 and BP4 in a Bayesian fashion by calculating the odds of pathogenicity for a variant above or below a chosen threshold based on the test set of variants. Such a calculation could also be used to modify the strength of the evidence code if it could be shown, for example, that variants above a particular threshold had moderate or strong odds of pathogenicity [17]. Because few confidently curated missense variants in *DICER1* currently exist, the VCEP was unable to establish cutoffs through a Bayesian approach [27] and instead selected ≥0.75 and <0.50 as the PP3 and BP4 cutoffs, respectively, based on the general REVEL use guidelines [26] and a good visual separation of 15 pathogenic and 27 benign variants. PP3 and BP4 may also be applied to splicing and noncoding variants based on the concordance of two splice predictors, MaxEntScan and SpliceAI. Until sufficient data are available to determine gene-specific splice predictor thresholds, standard MaxEntScan and SpliceAI thresholds should be used. PP3 should not be used in combination with PVS1.

#### 3.3.3. *BP7*

BP7 is intended for silent variants not predicted to impact splicing. BP4 must be applied as a prerequisite for BP7 consideration. For variants meeting BP4, any silent or intronic variant at +7 to -21 positions automatically qualifies for BP7. Noncoding variants outside the +7 to -21 intronic positions may have BP7 applied if the variant is the reference nucleotide in one or more primates and/or four or more mammalian species, indicating a lack of conservation of the nucleotide.

#### 3.3.4. *PM1*

Variation in critical gene regions or hotspot codons is considered moderate evidence of pathogenicity under PM1. *DICER1* has seven recognized hotspot codons: p.Ser1344, p.Glu1705, p.Asp1709, p.Asp1713, p.Gly1809, p.Asp1810, and p.Glu1813 [1, 24, 28]. Variation in these codons impairs the activity of the DICER1 RNase IIIb domain while leaving the IIIa cleavage domain intact. While variants in these hotspot codons are more commonly somatic in origin, they have been observed in a mosaic state and thus are still relevant for germline curation considerations [24, 29]. The VCEP decided it was appropriate to apply PM1 at a supporting level for missense variants affecting other residues within the RNase IIIb domain (p.Y1682–p.S1846).

#### 3.3.5. *PM4*

Similarly, the VCEP decided that in-frame protein length changes, considered moderate evidence of pathogenicity under PM4, were more likely to be pathogenic if located in the RNase IIIb domain [30, 31]. For this reason, PM4 can be applied at full moderate strength to in-frame indels within the RNase IIIb domain (p.Y1682–p.S1846) and at a supporting level to in-frame indels outside that domain. PM4 should not be applied to indels in repeat regions of *DICER1* (p.D606-p.D609; p.E1418-p.E1420; p.E1422-p.E1425).

#### 3.3.6. *PS1* and *PM5*

The PS1 and PM5 codes are intended for missense variants observed at an amino acid residue where the same (PS1) or a different (PM5) predicted amino acid change has been established as pathogenic. For both codes, the VCEP specified that the other variant must have reached a pathogenic classification (likely pathogenic does not suffice) by the *DICER1* VCEP and that splice effects should be ruled out by RNA data or concordance with MaxEntScan and SpliceAI. For PM5, the missense variant under investigation should have an equal or worse (i.e., higher) Grantham score than the other pathogenic variant [32]. The VCEP further expanded the scope of PS1 by allowing it to apply to noncanonical intronic nucleotide substitutions where a pathogenic splice site variant has been observed before if MaxEntScan and SpliceAI both predict an equal or greater splice impact for the variant under investigation. Because PS1, PM5, and PM1 are similar evidence types, they should not be applied together. The strongest evidence code should be used for variants meeting two or more of these codes. PM1 at supporting strength may be combined with PS1 or PM5.

### 3.4. Functional Data (PS3 and BS3)

#### 3.4.1. *PS3* and *BS3*

*In vivo* and *in vitro* functional studies provide another critical piece of evidence for variant curation under PS3 and BS3. The VCEP identified various types of functional evidence applicable to *DICER1* that can be applied at different strength levels. To apply PS3 at full strength, a patient-derived RNA assay must demonstrate an out-of-frame splicing impact or an in-frame splicing impact removing more than 10% (193 residues) of the protein or disrupting the RNase IIIb domain. If a variant also has PVS1_Strong applied, PS3 should be dropped to moderate application. PS3 can also be applied at a moderate level if RNA data demonstrates an in-frame splicing impact removing less than 10% of the protein and not affecting the RNase IIIb domain. Similarly, a patient-derived RNA assay demonstrating no splicing impact qualifies for BS3, though this should be observed in more than one patient to minimize the possibility of dropout. Another functional assay of utility for *DICER1* variant classification is an *in vitro* cleavage assay which assesses the ability of a DICER1 protein to generate 3p and 5p miRNAs [33]. Evidence of impaired or retained DICER1 cleavage function through such an assay may be used to apply PS3 or BS3, respectively, at a supporting level, provided that appropriate positive and negative controls were used. A higher strength level is not appropriate at this time as these assays are low-throughput and dependent on operator experience. PS3 cannot be applied at any strength if PVS1 is applied at full strength.

### 3.5. Clinical Data

#### 3.5.1. Phenotype (PS4 and PP4)

*(1) PS4*. The VCEP critically evaluated known DICER1*-*associated phenotypes; the specificity of these phenotypes for an underlying pathogenic germline *DICER1* variant was also considered. PS4 was initially intended to be an evidence code for variant-level case-control studies, with a reduced-strength option for rare variants observed in multiple affected patients but lacking statistically significant case-control studies [9]. The code has since evolved into a sophisticated proband-counting code with variable strength applications where affected, unrelated probands are allotted 0, 0.5, or 1 point each based on the specificity of their phenotypes, and the point total determines the PS4 strength application [12, 14]. The VCEP kept this framework in mind when considering the *DICER1* phenotypic spectrum.

A high-specificity phenotype deserving a full proband point should reflect a greater than 80% likelihood of an underlying pathogenic germline variant in the gene of interest; a moderate-specificity phenotype deserving a half proband point should reflect a 60-80% likelihood of an underlying causative germline variant [14]. Of the nearly 30 DICER1-associated phenotypes gathered from the literature [1, 5, 34] and panel members, few had published data on the frequency of underlying germline *DICER1* variants in unselected patient cohorts. Studies of pleuropulmonary blastoma [24] and pituitary blastoma [29] suggest greater than 80% specificity for an underlying pathogenic germline *DICER1* variant, while cystic nephroma [35] and Sertoli-Leydig cell tumors and gynandroblastoma [36] appear to fall in the 60-80% range. More recently, studies of primary intracranial sarcomas [37, 38] and multinodular goiter in young adults [39] suggest less than 60% specificity for germline *DICER1* variants.

Given the lack of large, unselected studies of these neoplasms, the VCEP elected to independently survey six clinical experts from the VCEP to categorize the phenotypes as high-specificity (much more likely than not to have a germline P/LP *DICER1* variant), moderate-specificity (more likely than not to have a germline P/LP *DICER1* variant), and low-specificity (less likely to have a germline P/LP *DICER1* variant). A consensus was reached if 5 or more of the experts agreed on the categorization. VCEP members discussed cases of disagreement and conservatively downgraded specificity. Certain phenotypes were considered so nonspecific (e.g., adult multinodular goiter, macrocephaly) that they were not deemed fit to qualify even for low specificity. The final agreed-upon designations are summarized in Table 2.

Using Table 3 as a guide, unrelated probands may be granted a full point on the basis of a high-specificity phenotype, two moderate-specificity phenotypes, a moderate-plus a low-specificity phenotype, or a moderate-specificity phenotype plus a family history of a high- or moderate-specificity phenotype in a first- or second-degree relative. If the last combination is used and that family also contributes to PP1 meiosis counting, only a half point should be counted to avoid double-counting segregation. A proband with only one moderate-specificity phenotype should be given a half point. Anything less specific is not granted any points. Points summed across unrelated probands indicate the strength application of PS4: supporting (1 to <2 points), moderate (2 to <4 points), or strong (≥4 points). PS4 should not be applied when a variant also has population data meeting BA1 or BS1 since a common variant may be present in a proband by chance. Additionally, PS4 should not be applied to a proband with another germline variant that could have reasonably contributed to the observed phenotype or whose tumor sequencing suggests sporadic tumorigenesis.

**Figure 1:**
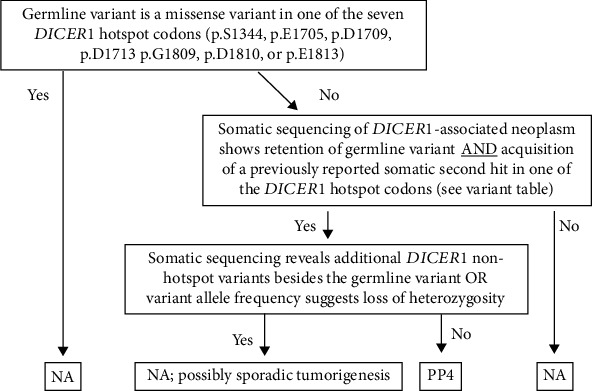
Flowchart for *DICER1-*specific PP4 code application.

*(2) PP4*. Considering PS4 proband counting, many VCEPs have discarded PP4, a code focused on patient phenotype and family history, as redundant. However, it has been recognized that PP4 may be utilized as a tumor phenotype code when appropriate [40]. With few exceptions, both benign and malignant *DICER1-*driven neoplasms follow a distinct modified two-hit hypothesis: one loss of function variant plus one variant selectively impairing the RNase IIIb domain function [2, 24, 41, 42]. In *DICER1-*related tumor predisposition, the germline variant is typically the loss of function, and the somatic second hit generally occurs in one of a handful of hotspot codons. Because this pattern is a hallmark of *DICER1-*driven neoplasms, the VCEP determined that evidence from somatic tumor sequencing of any *DICER1*-associated neoplasm, regardless of specificity, should lead to PP4 application if three conditions are met. First, the variant under investigation should not itself be in a *DICER1* hotspot codon. Second, in addition to the retention of the germline variant in the tumor, somatic sequencing should reveal a previously reported somatic second hit [1, 43, 44] as summarized in Supplementary Table 1. Finally, no additional non-hotspot *DICER1* variants or loss of heterozygosity should be revealed, as such a finding could reflect sporadic tumorigenesis. A flowchart simplifying the PP4 application is shown in Figure 1. A single observation of such evidence is sufficient for the PP4 application. Multiple observations cannot increase the code strength, as this would be considered proband counting. The VCEP will consider whether PP4 should be strengthened in future versions once a sufficient number of variants have been curated to allow for formal odds of pathogenicity calculations.

#### 3.5.2. Segregation Data (BS4 and PP1)

*(1) PP1 and BS4*. Variant segregation and lack of segregation with disease fall under PP1 and BS4, respectively. For counting PP1 meiosis, the *DICER1* VCEP adopted the same cutoffs used by other VCEPs [12–14] and informed by prior work [45, 46]. Namely, PP1 may be applied at supporting strength when 3 or 4 meioses are observed across one or more families, moderate strength when 5 or 6 meioses are observed across one or more families, and strong strength when seven or more meioses are observed across two or more families. Meioses are counted between phenotype-positive individuals with high-, moderate-, or low-specificity phenotypes as outlined in Table 2. PP1 was relaxed to include low-specificity phenotypes during the pilot, which improved its performance for pathogenic variants without resulting in excessive segregation counts. However, variant segregation with a single low-specificity phenotype (e.g., Wilms tumor) across multiple individuals is not sufficient for PP1 application. PP1 should not be applied when a variant also has population data meeting BA1 or BS1 since a common variant may appear to segregate with the disease by chance. BS4 may be applied if a proband has a phenotype-positive (must be high- or moderate-specificity), genotype-negative first-, second-, or third-degree relative. Genotype-positive and phenotype-negative individuals do not count toward BS4 but may be considered for BS2 (see 3.5.4).

#### 3.5.3. De Novo Data (PS2)

*(1) PS2*. The *DICER1* VCEP followed SVI recommendations for *de novo* criteria (https://clinicalgenome.org/working-groups/sequence-variant-interpretation/). Under the recommended framework, probands with *de novo* germline variants contribute 0, 0.25, 0.5, 1, or 2 points toward a *de novo* score based on the phenotype of the proband and whether parental relationships were confirmed (e.g., trio exome, maternity/paternity testing) or unconfirmed. Under this framework, a curator may apply either of the two *de novo* evidence codes originally proposed in the ACMG/AMP guidelines [9]. The *DICER1* VCEP elected to adopt PS2 as the sole *de novo* evidence code and to exclude PM6 as redundant, instead using PS2 at a lower evidence strength when maternity/paternity was unconfirmed. The proposed point combinations are summarized in Table 3, and phenotypes are organized in Table 2. Points summed across unrelated probands indicate the strength application of PS2: supporting (0.5 to <1 point), moderate (1 to <2 points), strong (2 to <4 points), or very strong (≥4 points).

#### 3.5.4. Allelic Data (BS2 and BP2)

*(1) BS2*. Because pathogenic *DICER1* variants have incomplete penetrance, the *DICER1* VCEP initially excluded BS2, which is considered benign evidence for a variant observed in a healthy adult. However, it became apparent during the pilot that a modified version of BS2 would be needed for multiple known benign variants to comfortably reach a benign classification. Based on a conservative neoplasm penetrance estimate of 10% in individuals aged 50-60 years with germline *DICER1* variants [22] and higher penetrance in females than males, the VCEP determined that an observation of 10 or more unrelated females, who have reached 50 years of age without a tumor diagnosis, should qualify for BS2_Supporting, provided that the ratio of BS2-eligible females to PS4-eligible probands is equal to or greater than 10 : 1. Similarly, since a strong evidence code can be thought of as equivalent to four supporting level codes [17], an observation of 40 or more unrelated females, who have reached 50 years of age without a tumor diagnosis, should qualify for BS2 at full strength, provided that the ratio of BS2-eligible females to PS4-eligible probands is equal to or greater than 40 : 1. In both cases, all females should come from a single source (e.g., from a single laboratory, database, clinical cohort, or publication) to eliminate the possibility of double counting. Additionally, since the homozygous loss of function variants in *DICER1* are thought to be embryonic lethal [47, 48], homozygous observations can also qualify for BS2 application. The *DICER1* VCEP allows BS2 to be applied at full strength if homozygosity is observed in two or more healthy individuals or in one healthy individual if homozygosity is confirmed by parental testing. BS2_Supporting may be applied if two or more observations of homozygosity are made in individuals lacking clinical information.

*(2) BP2*. In cases where an additional P/LP germline *DICER1* variant is found in a proband, BP2 may be applied if the P/LP variant is confirmed *in trans* with the variant under investigation. If the P/LP variant is *in cis* or in an unknown phase, three such observations are required for BP2 application, and the probands must not all carry the same P/LP variant. Similar to PS1 and PM5, the cooccurring P/LP variant must be classified by the *DICER1* VCEP.

### 3.6. Evidence Code Combinations

Initially, the VCEP followed the originally recommended evidence code combinations [9] and stated that a single supporting evidence code should not be considered conflicting evidence if a clinically meaningful classification would otherwise be reached. However, the original combinations were not flexible enough to account for some of the combinations in round 1 of the pilot (e.g., 6 supporting pathogenic codes), and limitations with regard to resolving complex conflicting evidence code combinations are apparent. For those reasons, the VCEP pivoted to a flexible, modified Bayesian point approach for all evidence code combinations [18] for the final pilot curations. In this approach, supporting, moderate, strong, and very strong evidence codes are weighted at one, two, four, and eight points, respectively, with pathogenic evidence weighted positively and benign evidence weighted negatively. A sum of the points results in the final classification as outlined in Table 4.

### 3.7. Pilot

The VCEP tested the proposed evidence code specifications on 40 *DICER1* variants as described in the Methods. The pilot results, including the evidence codes applied, are summarized in Table 5. To improve performance, the VCEP modified PP1, BS2, and the method for evidence code combinations as described above between round 1 and round 2 of the pilot. The changes implemented between the initial and final rounds of pilot classifications led to stronger variant classifications (i.e., more pathogenic or more benign) in nine variants (22.5%), including five variants which shifted from VUS to LB or LP.

Final VCEP classifications were clinically meaningful for 82.5% (33/40) of the pilot variants. Concordance for known P/LP and known B/LB pilot variants was 100% (14 of 14 P/LP and 12 of 12 B/LB). Pilot variants with conflicting or uncertain classifications in ClinVar reached 50% (7/14) resolution, with four variants reaching LB and three reaching LP.

## 4. Discussion

Under the ClinGen framework, the *DICER1* VCEP developed and piloted *DICER1*-specific sequence variant curation guidelines. These guidelines performed very well on a set of pilot variants, with more than 80% of the pilot variants receiving a clinically meaningful classification. Furthermore, the pilot demonstrated that the guidelines could be interpreted and applied consistently by curators and that internal data sharing can be effectively integrated into the curation process. The pilot variants and evidence summaries have been submitted to ClinVar as three-star submissions [49]. Additional curation details for those variants are also available on the ClinGen Evidence Repository (https://erepo.clinicalgenome.org/evrepo/).

Past challenges in curating *DICER1* missense variants have been recognized and even cited as a reason to exclude *DICER1* from the ACMG secondary findings list [50]. The success of our guidelines in clarifying *DICER1* variant classification not only implies fewer patients will be faced with VUS results in the future but also reduces this barrier for future reconsideration of *DICER1* for the ACMG secondary findings list.

The VCEP will continue to meet regularly to further variant curation progress and submit classifications for public use. Variants will be prioritized by ClinVar classification (conflicting interpretations or VUS by multiple submitters) and by request. ClinVar currently contains ~5,000 *DICER1* variant entries, including ~150 with conflicting interpretations and ~860 VUS by multiple submitters. Variant interpretations will be submitted to ClinVar within 30 days of VCEP approval. The VCEP will recurate variants classified as LP or VUS every two years to assess whether additional evidence is available. Medically significant discrepancies (i.e., P/LP vs. VUS/LB/B) between a VCEP submission and a more recent ClinVar submitter will be reviewed and updated as appropriate within six months of the discrepant submission. Other discrepancies (i.e., VUS vs. LB/B) will be reviewed within two years.

Due to the characteristic signature of DICER1 somatic mutations, the *DICER1* VCEP chose to use somatic tumor testing as supporting evidence (PP4) [40]. The DICER1 VCEP is the first VCEP within the ClinGen Hereditary Cancer Clinical Domain to use somatic tumor testing to inform the PP4 application, providing a model for other VCEPs.

As more is learned and published on the *DICER1* gene and the phenotypic consequences of its pathogenic variation, the VCEP will reevaluate the proposed guidelines and consider updates for future versions of the guidelines. For example, the phenotypic spectrum of the disorder may expand, or the specificity of certain phenotypes may need to be adjusted. Additionally, as more *DICER1* variants are curated, the VCEP can revisit the odds of pathogenicity calculations for various evidence codes such as PP4 tumor phenotype evidence or PP3 and BP4 *in silico* predictor cutoffs and modify the strength of the evidence codes as appropriate. Any modifications to evidence specifications will be submitted to the SVI for approval and made publicly available on the ClinGen website (https://clinicalgenome.org/affiliation/50050/) as a resource for others curating *DICER1* variants.

## 5. Conclusions

The *DICER1-*specific sequence variant curation guidelines developed by the ClinGen *DICER1* VCEP show promising results on a pilot set of 40 variants, with 80% reaching clinically meaningful classifications. Consistent utilization of these guidelines may reduce the number of variants of uncertain significance returned to patients undergoing *DICER1* sequencing. Future refinement of these guidelines over time is expected to further improve the clinical utility of variant classification.

## Figures and Tables

**Table 1:** Summary of *DICER1*-specific specifications of the ACMG/AMP variant curation guidelines.

Original ACMG/AMP evidence codes		*DICER1* specifications
Criteria	Criteria description	
PVS1	Null variant in a gene where loss of function is a known mechanism of disease.	Follow SVI-approved decision tree (Figure S1) with *DICER1*-specific modifications: (i) NMD cutoff: p.Pro1850 (ii) In-frame exon(s): 5,10, 15, 18, 22 (iii) Noncoding exon(s): 1 (iv) Final exon: 27 (v) 10% of protein = 193 amino acids (vi) No criteria applied for disruption of start codon p.M1

PS1	Same amino acid change as a previously established pathogenic variant regardless of nucleotide change.	Other variant must be interpreted as pathogenic by the *DICER1* VCEP. Likely pathogenic changes do not apply. Same amino acid change: must confirm there is no difference in splicing. Noncanonical intronic splicing variants at same nucleotide: should have equal or worse splicing impact. Caveat: do not apply PM1 (full strength) or PM5 if PS1 is applied.

PS2	*De novo* (proven or assumed) in a patient with disease and no family history.	Follow the point structure outlined in the manuscript and summarized in Table 3. PS2_Very strong: ≥4 points PS2: ≥2 but less than 4 points PS2_Moderate: ≥1 but less than 2 points PS2_Supporting: ≥0.5 but less than 1 point

PS3	Well-established *in vitro* or *in vivo* functional studies supportive of a damaging effect.	PS3: patient-derived RNA assay demonstrates splicing impact that is out-of-frame OR in-frame with ≥193 residues affected OR disrupting the RNase IIIb domain. (downgrade to PS3_Moderate if PVS1_Strong is also met) PS3_Moderate: patient-derived RNA assay demonstrates splicing impact that is in-frame and disrupts <193 residues, leaving the RNase IIIb domain intact. PS3_Supporting: *in vitro* cleavage assay with positive and negative controls demonstrates severely reduced capacity to produce 5p and/or 3p miRNA from pre-miRNA. Caveat: do not apply PS3 at any strength if PVS1 is applied at full strength.

PS4	The prevalence of the variant in affected individuals is significantly increased compared with the prevalence in controls.	Follow the point structure outlined in the manuscript and summarized in Table 3. PS4: ≥4 points PS4_Moderate: 2-3.5 points PS4_Supporting: 1-1.5 points Caveats: do not apply for variants that meet BA1 or BS1. Do not apply proband points for an individual who has another germline variant that could have reasonably contributed to the phenotype or whose tumor sequencing suggests sporadic tumorigenesis.

PM1	Located in a mutational hotspot and/or critical and well-established functional domain.	PM1: putative missense variants at residues affecting RNase IIb domain metal ion-binding (p.S1344, p.E1705, p.D1709, p.D1713, p.G1809, p.D1810, and p.E1813). PM1_Supporting: putative missense variants affecting other residues in the RNase IIIb domain (p.Y1682–p.S1846). Caveat: the full strength rule cannot be applied with PS1 or PM5.

PM2	Absent/rare from controls in an ethnically matched cohort population sample.	This rule code is only applicable at a supporting level. PM2_Supporting: allele frequency < 0.000005 across gnomAD (noncancer) with no more than one allele in any subpopulation and at least 20x coverage.

PM3	For recessive disorders, detected in trans with a pathogenic variant.	N/A–DICER1 syndrome follows autosomal dominant inheritance

PM4	Protein length changes due to in-frame deletions/insertions in a nonrepeat region or stop-loss variants.	PM4: in-frame indels with a residue within the RNase IIIb domain (p.Y1682–p.S1846). PM4_Supporting: in-frame indels outside of the RNase IIIb domain (p.Y1682–p.S1846) and repeat regions (p.D606-p.D609; p.E1418-p.E1420; p.E1422-p.E1425).

PM5	Missense change at an amino acid residue where a different missense change determined to be pathogenic has been seen before.	Other variant must be interpreted as pathogenic by the *DICER1* VCEP. Likely pathogenic changes do not apply. The variant under assessment should have an equal or worse Grantham score. MaxEntScan and SpliceAI should demonstrate no splicing impact. Caveat: do not apply with PS1 or with full-strength PM1.

PM6	Assumed de novo, but without confirmation of paternity and maternity.	N/A–Considered redundant after PS2 modifications

PP1	Cosegregation with disease in multiple affected family members.	Phenotype-positive individuals should have high, moderate, or low-specificity phenotypes (see Table 2). PP1_Strong: ≥7 meioses across ≥2 families PP1_Moderate: 5 or 6 meioses across ≥1 family PP1: 3 or 4 meioses across ≥1 family Caveats: do not apply for variants that meet BA1 or BS1. Segregation with a single low-specificity phenotype across multiple individuals does not fulfill PP1.

PP2	Missense variant in a gene that has a low rate of benign missense variation and where missense variants are a common mechanism of disease.	N/A–while DICER1 does meet recommended cutoff for missense constraint, z score of ≥3.09 established by the SVI (4.23 on gnomAD), the DICER1 VCEP recommends this rule not be used due to the presence of various missense variants throughout the gene that are clinically interpreted as benign (9) or likely benign (30) in ClinVar.

PP3	Multiple lines of computational evidence support a deleterious effect on the gene or gene product.	Missense variants: REVEL score ≥ 0.75 OR concordance of MaxEntScan and SpliceAI for prediction of splice impact. Splicing variants: concordance of MaxEntScan and SpliceAI for prediction of splice impact. Caveat: do not apply in combination with PVS1.

PP4	Patient's phenotype or family history is highly specific for a disease with a single genetic etiology.	Tumor testing of a neoplasm within the DICER1 syndrome phenotypic spectrum in a proband with the germline variant under assessment reveals the following: (i) A previously reported somatic second hit in a *DICER1* hotspot codon (Table S1) AND (ii) Retention of the germline variant under assessment See Figure 1 for a PP4 application flowchart. Caveats: PP4 cannot be applied to germline curation of variants in the *DICER1* hotspot codons (p.S1344, p.E1705, p.D1709, p.D1713, p.G1809, p.D1810, or p.E1813). PP4 cannot be applied if tumor testing reveals any additional *DICER1* non-hotspot variant(s).

PP5	Reputable source recently reports variant as pathogenic but the evidence is not available to the laboratory to perform an independent evaluation.	N/A per published SVI guidance

BA1	Allele frequency is above 5% in Exome Sequencing Project, 1000 Genomes, or ExAC.	Allele frequency > 0.003 (0.3%) in gnomAD (noncancer) subpopulations. Subpopulations must have >2,000 alleles tested and a minimum of 5 alleles present.

BS1	Allele frequency is greater than expected for disorder.	Allele frequency > 0.0003 (0.03%) in gnomAD (noncancer) subpopulations. Subpopulations must have >2,000 alleles tested and a minimum of 5 alleles present.

BS2	Observed in a healthy adult.	BS2: 40+ unrelated females from a single source have reached age 50 without a tumor diagnosis (ratio of BS2-eligible females to PS4-eligible probands must be ≥40 : 1) OR 2+ observations of homozygosity in healthy individuals OR 1+ observation(s) of homozygosity in a healthy individual with status confirmed by parental testing. BS2_Supporting: 10+ unrelated females from a single source have reached age 50 without a tumor diagnosis (ratio of BS2-eligible females to PS4-eligible probands must be ≥10 : 1) OR 2+ observations of homozygosity in individuals lacking clinical information.

BS3	Well-established *in vitro* or *in vivo* functional studies show no damaging effect on protein function.	BS3: for intronic or synonymous variants, ≥2 observations of no splicing impact via patient-derived RNA assay. BS3_Supporting: *in vitro* cleavage assay with positive and negative controls demonstrates retained ability to produce 5p and 3p miRNA from pre-miRNA.

BS4	Lack of segregation in affected members of a family.	Variant observed in at least one phenotype-positive (must be high- or moderate-specificity phenotype; see Table 2), genotype-negative 1st, 2nd, or 3rd degree relative(s) of the proband. This rule does not apply to phenotype-negative, genotype-positive family members.

BP1	Missense variant in gene where primarily truncating variants cause disease.	N/A–Truncating variants account for only a portion of disease-causing variants

BP2	Observed in trans with a pathogenic variant for a fully penetrant dominant gene/disorder or observed in cis with a pathogenic variant in any inheritance pattern.	Observed *in trans* with a pathogenic or likely pathogenic *DICER1* variant (phase confirmed) in at least 1 individual OR observed *in cis* and/or phase unknown in at least 3 individuals, at least 2 of whom carry unique pathogenic/likely pathogenic *DICER1* variants. This rule code can only be used to compare variants asserted as pathogenic by the ClinGen *DICER1* VCEP. Homozygous cases are not relevant for BP2 and should instead contribute to BS2.

BP3	In-frame deletions/insertions in a repetitive region without a known function.	N/A

BP4	Multiple lines of computational evidence suggest no impact on gene or gene product.	Missense variants: REVEL score < 0.50 AND concordance of MaxEntScan and SpliceAI predicting no splice effects. Synonymous, intronic, and noncoding variants: concordance of MaxEntScan and SpliceAI predicting no splice effects.

BP5	Variant found in a case with an alternate molecular basis for disease.	N/A–given the broad spectrum of DICER1-related neoplasms and the general lack of evidence of other high-penetrance germline variants that could account for such neoplasms (except perhaps for some low-specificity phenotypes), this rule should not be used at this time.

BP6	Reputable source recently reports variant as benign but the evidence is not available to the laboratory to perform an independent evaluation.	N/A per published SVI guidance

BP7	A synonymous (silent) variant for which splicing prediction algorithms predict no impact to the splice consensus sequence nor the creation of a new splice site AND the nucleotide is not highly conserved.	This rule applies to silent variants and intronic variants at or beyond +7 to -21 positions. For other intronic or noncoding variants, BP7 may be applied if the variant is the reference nucleotide in ≥1 primate and/or ≥4 mammalian species. Caveat: BP7 cannot be applied unless BP4 is also met.

**Table 2:** DICER1 syndrome phenotypes grouped by specificity. For use with the following evidence codes: PS4, PS2, PP1, PP4, and BS4.

Specificity	Phenotypes
High-specificity (much more likely than not to have germline P/LP DICER1)	Pleuropulmonary blastoma (PPB) (including type 1r)
	Pituitary blastoma
	Anaplastic renal sarcoma
	Ciliary body medulloepithelioma
	Cystic nephroma (<18 yrs)
	Embryonal rhabdomyosarcoma (ovarian)
	Embryonal rhabdomyosarcoma (cervix)

Moderate-specificity (more likely than not to have germline P/LP DICER1)	Differentiated thyroid cancer and/or multinodular goiter (<18 years)
	Nasal chondromesenchymal hamartoma
	Ovarian Sertoli-Leydig cell tumors
	Ovarian sex-cord stromal tumor of mixed type (specifically, gynandroblastoma)

Low-specificity (less likely to have DICER1)	Nonparasitic liver cysts (childhood)
	Wilms tumor
	Pineoblastoma
	Cerebral sarcoma
	Lung cysts (<18 yrs)

^∗∗^for PP4 use ONLY^∗∗^ additional neoplasms of very low or undetermined specificity	Thyroid neoplasms (any age)
	Sarcomas
	Juvenile hamartomatous polyps
	Primitive neuroectodermal/neuroepithelial neoplasms
	Infantile cerebellar embryonal tumors
	Fetal lung adenocarcinoma

**Table 3:** Points per proband that can be applied toward PS2 and/or PS4 application based on proband phenotype and confirmed or assumed *de novo* status. Modified from “SVI Recommendation for *De Novo* Criteria (PS2 and PM6)”–Version 1.0.

Phenotypic consistency	Points per proband			Proband phenotype (see Table 2)
	PS2		PS4	
	Confirmed	Assumed		
Phenotype highly specific for gene	2	1	1	(i) ≥1 high OR (ii) ≥2 moderate OR (iii) 1 moderate AND (a) ≥1 low OR (b) High or moderate in 1st- or 2nd-degree relative (unless known not to carry variant)^†^

Phenotype consistent with gene but not highly specific	1	0.5	0.5	(iv) 1 moderate

Phenotype consistent with gene but not highly specific and high genetic heterogeneity^‡^	0.5	0.25	0	(v) ≥1 low

^†^If PP1 is applied and the proband's family contributed to the PP1 meiosis count, use IV (1 moderate) instead of IIIb to avoid double counting family history. ^‡^Maximum allowable value of 1 may contribute to overall PS2 score to avoid counting multiple probands with only low-specificity phenotypes.

**Table 4:** Points system for classifying *DICER1* germline variants. Supporting, moderate, strong, and very strong codes receive 1, 2, 4, and 8 points, respectively, with pathogenic evidence codes in the positive direction, and benign evidence codes in the negative direction. Adapted from [18] (PMID: 32720330).

Category	Point ranges
Pathogenic	≥10
Likely pathogenic	6 to 9
Uncertain	0 to 5
Uncertain with caveat^†^	-1
Likely benign	-2 to -6
Benign	≤ -7

^†^A final point value of -1 may be overridden to likely benign only in cases where PM2_Supporting is applied AND no other pathogenic evidence codes are applied (e.g. BP4, BP7, and PM2_Supporting).

**Table 5:** Classification of 40 germline *DICER1* variants during the pilot phase of the *DICER1-*specific ACMG/AMP variant curation guidelines. Round 1 and round 2 criteria reflect criteria from the preliminary and finalized guidelines, respectively.

*DICER1* variant	ClinVar ID	ClinVar classifications^†^	Round 1 criteria applied^‡^	Initial classification	Round 2 criteria added^‡^	Points	Final classification^§^
c.4748T>G (p.L1583R)	4468	P/LP	PM2_P, PP3, PS3_P, PS4_P, PP1	VUS	PP1 ⟶ PP1_M	6	**LP**
c.5125G>A (p.D1709N)	690480	P	PM2_P, PP3, PM1, PS3_P, PS4_M, PS2_VS	P		15	P
c.5441C>T (p.S1814L)	412119	P/LP	PM2_P, PP3, PM1_P, PS3_P, PS4, PP4, PP1_S	P		13	P
c.5465A>T (p.D1822V)	254350	P/LP	PM2_P, PP3, PM1_P, PS3_P, PS4_M	LP		6	LP
c.5138A>T (p.D1713V)	690454	P/LP	PM2_P, PP3, PM1, PS3_P, PS2_P	LP		6	LP
c.5104C>T (p.Q1702^∗^)	254344	P	PM2_P, PVS1, PS4_P	P		10	P
c.1408G>T (p.E470^∗^)	254287	P	PM2_P, PVS1, PS4_P, PP4	P		11	P
c.3019C>T (p.Q1007^∗^)	429113	P	PM2_P, PVS1, PS4_P, PP4	P		11	P
c.1880_1883del (p.I627fs)	254298	P	PM2_P, PVS1, PS4_P, PP1	P		11	P
c.878_881del (p.R293fs)	254355	P	PM2_P, PVS1, PS4_P, PP4	P		11	P
c.2650+1G>T	254310	P	PM2_P, PVS1, PS4_M	P		11	P
c.1907+1G>A	429148	P/LP	PM2_P, PVS1, PS4_P, PM6	P		12	P
c.2988−1G>T	429116	P	PM2_P, PVS1, PS4_M, PS2	P		15	P
c.5479del (p.1827fs)	477261	P/LP	PVS1, PM2_P, PS4_P	P		10	P
c.2614G>A (p.A872T)	133967	B/LB	BS1, BP4, BS3_P	LB	BS2	-10	**B**
c.4910C>T (p.S1637L)	242127	B/LB	BS1, BP4	LB	BS2	-9	**B**
c.3828T>A (p.D1276E)	794388	LB	BS1, BP4	LB		-5	LB
c.3428T>C (p.L1143P)	220594	B/LB	BS1, BP4	LB	BS2	-9	**B**
c.1825G>T (p.D609Y)	133965	B/LB	BA1 (BS1, BP4)	B	BS2	NA	B
c.884C>G (p.S295C)	242151	B/LB	BA1 (BP4)	B	BS2	NA	B
c.59C>T (p.A20V)	133964	B/LB	BS1, BP4	LB	BS2	-9	**B**
c.5052C>G (p.L1684=)	242128	LB	BS1, BP4, BP7	LB		-6	LB
c.2808T>C (p.Y936=)	751425	LB	BP4, BP7	LB		-4	LB
c.4647C>T (p.H1549=)	417113	B/LB	BP4, BP7	LB	BS2	-6	LB
c.5521C>T (p.L1841=)	477262	LB	PM2_P, BP4, BP7	LB		-1	LB
c.3269+14G>A	315107	B/LB	BP4, BP7	LB	BS2	-6	LB
c.5096−12G>A	580203	VUS	PS3, PS4_M, PP4, PM2_P	LP		8	LP
c.1722_1724del (p.E574del)	566588	VUS	PM2_P, PM4_P	VUS		2	VUS
c.2651−4T>G	543697	Conflicting: LB(1); VUS(1)	BP4, BP7	LB		-2	LB
c.238G>T (p.E80^∗^)	649946	Conflicting: P(1); VUS(1)	PVS1, PM2_P	LP		9	LP
c.5276A>G (p.K1759R)	133974	Conflicting: LB(1); VUS(1)	BP4, PM1_P	VUS	BS2	-4	**LB**
c.5107C>T (p.R1703C)	242130	VUS	PP3, PM1_P, PM2_P	VUS		3	VUS
c.4178_4180dup (p.N1393dup)	242100	VUS	PM4_P	VUS	BS2_P	0	VUS
c.^∗^5G>A	315099	Conflicting: LB(1); VUS(2)	BP4	VUS	BS2_P	-2	**LB**
c.5563C>T (p.R1855^∗^)	947388	VUS	PVS1_M, PM2_P	VUS		3	VUS
c.4024C>T (p.R1342C)	412120	VUS	PM2_P	VUS		1	VUS
c.1907+3A>T	820285	Conflicting: LB(1); VUS(1)	PM2_P; BS3	VUS	Points	-3	**LB**
c.2T>C (p.M1T)	242076	VUS	PM4_P	VUS		1	VUS
c.5422A>G (p.M1808V)	477260	VUS	PM1_P	VUS		1	VUS
c.2642T>C (p.L881P)	690445	VUS	PP3, PP4, PM2_P, PS3_P, PS4_P	VUS	PP1, points	6	**LP**

^†^ClinVar classifications were pulled in November 2020 with the exception of p.D1713V and p.Leu1827fs due to a known incongruence between one laboratory's ClinVar submissions (VUS) and internal classifications (LP) at the time of the data pull. ^‡^Modified strength levels are denoted with an underscore followed by a P, M, S, or VS, denoting supporting, moderate, strong, or very strong strength. ^§^Classifications that changed between round 1 and round 2 are in bold text.

## Data Availability

The variant classifications made during this effort have been published in ClinVar (https://www.ncbi.nlm.nih.gov/clinvar/), and the curated evidence collected has been made publicly available through the ClinGen Evidence Repository (https://erepo.clinicalgenome.org/evrepo/). Some detailed internal patient-level data is not publicly available for ethical and privacy reasons.

## Supplementary Materials

Supplementary 1 Supplementary Table 1; *DICER1* hotspot variants previously reported as somatic second hits.

Supplementary 2 Supplementary Figure 1: flowchart for *DICER1-*specific PVS1 code application. Modified from [25] (PMID 30192042).
