# Supplementary material for: Specifications of the ACMG/AMP Variant Classification Guidelines for Germline DICER1 Variant Curation
Source: Hum Mutat. 2023 Mar 29;2023:9537832. doi: 10.1155/2023/9537832 (PMC10713350; doi:10.1155/2023/9537832)
Supplement: Supplementary 1 — Supplementary Table 1; DICER1 hotspot variants previously reported as somatic second hits. [file 9537832.f1.docx]

**Supplementary Table 1**. *DICER1* hotspot variants previously reported as somatic second hits.

|  | WT | Alternate |
| --- | --- | --- |
| 1344 | **Ser (S)** | Leu (L) |
| 1705 | **Glu (E)** | Asp (D), Gln (Q), Lys (K), Val (V) |
| 1709 | **Asp (D)** | Asn (N), Glu (E), Gly (G), Tyr (Y), Val (V) |
| 1713 | **Asp (D)** | Val (V) |
| 1809 | **Gly (G)** | Arg (R), Glu (E), Trp (W) |
| 1810 | **Asp (D)** | Asn (N), Gly (G), His (H), Tyr (Y), Val (V) |
| 1813 | **Glu (E)** | Ala (A), Asp (D), Gln (Q), Gly (G), Lys (K), Val (V) |
