# Supplementary figures and images for: Specifications of the ACMG/AMP Variant Classification Guidelines for Germline DICER1 Variant Curation
Source: Hum Mutat. 2023 Mar 29;2023:9537832. doi: 10.1155/2023/9537832 (PMC10713350; doi:10.1155/2023/9537832)

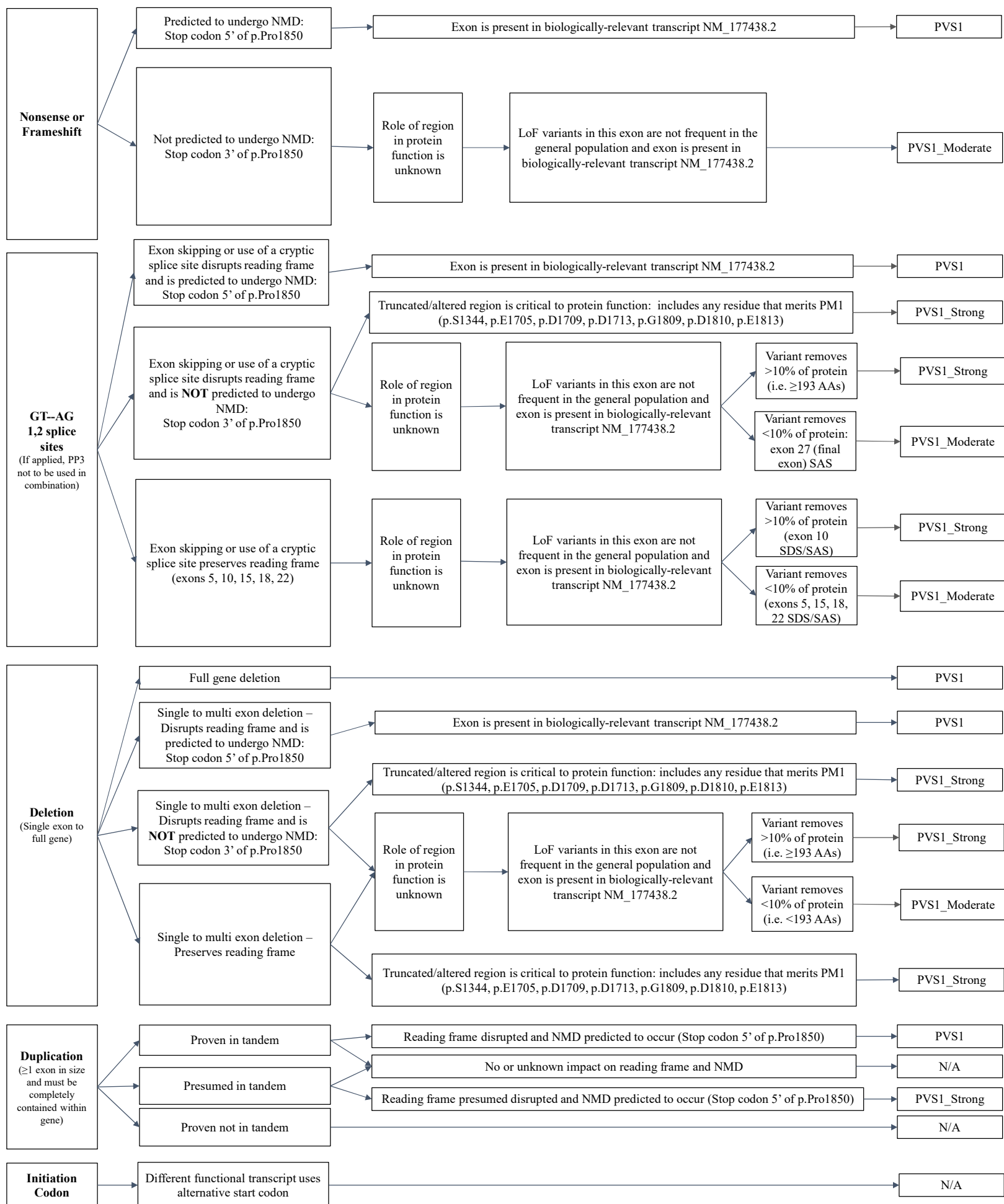

Supplement: Supplementary 2 — Supplementary Figure 1: flowchart for DICER1-specific PVS1 code application. Modified from [25] (PMID 30192042). [file 9537832.f2.pdf]
